# Air Quality in the Harbin-Changchun Metropolitan Area in Northeast China: Unique Episodes and New Trends

**DOI:** 10.3390/toxics9120357

**Published:** 2021-12-17

**Authors:** Yulong Wang, Youwen Sun, Gerong Zhao, Yuan Cheng

**Affiliations:** 1 State Key Laboratory of Urban Water Resource and Environment, School of Environment, Harbin Institute of Technology, Harbin 150090, China; yulongwang1994@hotmail.com (Y.W.); 21S129126@stu.hit.edu.cn (G.Z.); 2 Key Laboratory of Environmental Optics and Technology, Anhui Institute of Optics and Fine Mechanics, Chinese Academy of Sciences, Hefei 230031, China

**Keywords:** particulate matter, air pollution, northeast china, spatiotemporal variations

## Abstract

Because of the unique geographical, climate, and anthropogenic emission characteristics, it is meaningful to explore the air pollution in the Harbin-Changchun (HC) metropolitan area. In this study, the Air Quality Index (AQI) and the corresponding major pollutant were investigated for the HC cities, based on the air quality data derived from the China National Environmental Monitoring Center. The number of days with the air quality level of “good” gradually increased during recent years, pointing to an improvement of the air quality in HC. It was also found that ozone, a typical secondary pollutant, exhibited stronger inter-city correlations compared to typical primary pollutants such as carbon monoxide and nitrogen dioxide. In addition, for nearly all the HC cities, the concentrations of fine particulate matter (PM_2.5_) decreased substantially in 2020 compared to 2015. However, this was not the case for ozone, with the most significant increase of ozone observed for HC’s central city, Harbin. This study highlights the importance of ozone reduction for further improving HC’s air quality, and the importance of agricultural fire control for eliminating heavily-polluted and even off-the-charts PM_2.5_ episodes.

## 1. Introduction

Air pollution has been paid more and more attention by many studies due to its association with a variety of adverse health effects [1,2,3,4,5,6]. To improve air quality, the Chinese government formulated the new air quality standard (GB3095-2012) in 2012, issued the “Air Pollution Prevention and Control Action Plan” in 2013, and made the “Three-year Action Plan aims for Blue Skies” in 2018 [5,7,8,9,10]. China’s Ministry of Environmental Protection (MEP) and local environmental protection bureaus have established a relatively complete ground detection system [1,5,10]. The six criteria air pollutants (Particulate Pollutants; PM_2.5_ and PM_10_. Gaseous Pollutants; SO_2_, NO_2_, CO, and O_3_) can be continuously monitored by ground-based monitoring stations after 2013 and the open-access data can be obtained at the MEP [2,4,9]. According to China’s action plans, local governments began to implement different control policies for various sources of air pollutants, such as coal burning, road traffic, and open biomass burning (OBB) [5,10,11]. Air pollution in economically developed urban agglomerations such as the Beijing Tianjin Hebei (BTH), Yangtze River Delta (YRD), and Pearl River Delta (PRD) regions has improved significantly due to the government’s action plans [4,12,13,14,15,16,17]. With in-depth research on air pollution, scholars began to study air pollution in different regions of China to fill the gaps in this region, such as the Harbin-Changchun (HC) region [9,18,19,20,21,22,23].

HC is located in the Northeast of China [8]. Compared with other study areas, HC is unique in that it has experienced a heating season of up to half a year [9,22]. Due to the low temperature in winter (~−20 °C), the air pollutant components in this region have their unique regional characteristics [7]. The first feature in HC is the heavy use of fossil fuels, especially coal which is the primary energy source during the six-month heating period in urban areas [21]. The second feature is that heating in rural areas requires a large number of crop straws which also sends some air pollutants into the atmosphere [10]. Furthermore, the crop residue was usually burned directly by farmers to ensure the next spring tillage, which made a large number of air pollutants released into the atmosphere [24,25]. The phenomenon of OBB in HC is very common and has been investigated by many studies [11,22,23,26,27,28,29,30]. Because of the unique geographical, climate, and anthropogenic characteristics, it is very meaningful to study the composition of air pollutants in HC [31,32].

In this work, we used the six criteria air pollutants in 11 cities of HC from 2015 to 2020 obtained from MEP to calculate the Air Quality Index (AQI) and the major pollutants according to the HJ633-2012. The air pollution situation in HC is discussed according to the calculated data (AQI and the major pollutants). This paper also discussed whether the annual average of the six criteria air pollutants had reached the Grade II standards. In addition, the influences of OBB and dust weather on six criteria air pollutants in HC were discussed by using the characteristic ratios of PM_2.5_/SO_2_ and PM_10_/PM_2.5_. The correlation between 11 cities and the coordinated control of PM_2.5_ and O_3_ are discussed in the last part of the paper.

## 2. Materials and Methods

### 2.1. Study Areas

The Harbin-Changchun (HC) megalopolis, located in the Northeast of China, is the only national-level city cluster with an exceeding six months’ heating period. The HC consists of 11 cities, of which Harbin (HRB), Daqing (DQ), Qiqihar (QQHR), Suihua (SH), and Mudanjiang (MDJ) belong to Heilongjiang Province, and Changchun (CC), Jilin (JL), Siping (SP), Liaoyuan (LY), Songyuan (SY), and Yanbian (YB) belong to Jilin Province. Inner Mongolia and Mongolia are located in the northwest of HC, and the northeast side of the HC is connected to Russia.

### 2.2. Air Quality Data

The concentrations data of the six criteria air pollutants (PM_2.5_, PM_10_, SO_2_, CO, NO_2_, and O_3_-8 h) were downloaded from the publishing website of China Air Quality Online Monitoring and Analysis Platform (https://www.aqistudy.cn/, accessed on 16 December 2021). The locations of 58 monitoring stations in HC can be found in previous studies [2]. According to the GB3095-2012 (https://www.mee.gov.cn/ywgz/fgbz/bz/bzwb/dqhjbh/dqhjzlbz/201203/t20120302_224165.shtml, accessed on 16 December 2021), the O_3_-8 h refers to the arithmetic mean of the average concentration for 8 consecutive hours, also known as the 8-h moving average. The detection methods of the six criteria air pollutants have been stipulated in China Environmental Protection Standards HJ655-2013 (http://www.mee.gov.cn/ywgz/fgbz/bz/bzwb/jcffbz/201308/t20130802_256855.shtml, accessed on 16 December 2021) and HJ193-2013 (http://www.mee.gov.cn/ywgz/fgbz/bz/bzwb/jcffbz/201308/t20130802_256856.shtml, accessed on 16 December 2021) which was also displayed in Supplementary Material S1.

### 2.3. The Calculate of Air Quality Index (AQI) and Major Pollutant

According to the HJ633-2012 (http://www.mee.gov.cn/ywgz/fgbz/bz/bzwb/jcffbz/201203/t20120302_224166.shtml, accessed on 16 December 2021), the Air Quality Index (AQI) can be calculated according to the Equations (1) and (2). In Equation (1), the IAQI_P_ represents the individual air quality index (IAQI) of pollutant item P. The C_P_ represents the mass concentration of pollutant item P. The BP_Hi_ and BP_Lo_ represent the highest and lowest values of the concentration interval with C_P_ in Table 1, respectively. The IAQI_Hi_ and IAQI_Lo_ represent the IAQI values corresponding to BP_Hi_ and BP_Lo_ in Table 1, respectively.

(1) $${\mathrm{IAQI}}_{P}=\frac{{\mathrm{IAQI}}_{\mathrm{Hi}}-{\mathrm{IAQI}}_{\mathrm{Lo}}}{{\mathrm{BP}}_{\mathrm{Hi}}-{\mathrm{BP}}_{\mathrm{Lo}}}(C_{P}-{\mathrm{BP}}_{\mathrm{Lo}})+{\mathrm{IAQI}}_{\mathrm{Lo}}$$

After IAQI of each pollutant is calculated, the value of AQI is taken from the maximum value of each IAQI, the equation is shown in Equation (2).

AQI = max {IAQI_1_, IAQI_2_, IAQI_3_, ∙∙∙, IAQI_n_} (2)

The major pollutant refers to the pollutant with the largest IAQI when AQI is greater than 50. The pollutants with IAQI greater than 100 are non-attainment standard pollutants. The details of the calculation method for AQI can also be found in Wang et al. (2014) [1].

## 3. Results

### 3.1. Overview of Air Pollutants

The Air Quality Index (AQI) of 11 cities in HC was calculated according to the HJ633-2012. The air quality is divided into six levels (good, moderate, lightly polluted, moderately polluted, heavily polluted, and severely polluted) corresponding to the range of AQI (0–50, 51–100, 101–150, 151–200, 200–300, and >300). Besides, the Chinese Ambient Air Quality Standards (GB3095-2012) stipulates the Grade I standards and Grade II standards for the region I (nature reserves, scenic spots, and other areas requiring special protection) and region II (residential areas, commercial traffic, and mixed residential areas, cultural areas, industrial areas, and rural areas), respectively. The Grade I standard for PM_2.5_, PM_10_, SO_2_, and NO_2_ is 15, 40, 20, and 40 µg/m^3^, respectively. The Grade II standard for PM_2.5_, PM_10_, SO_2_, and NO_2_ is 35, 70, 60, and 40 µg/m^3^, respectively. There is no annual average standard for CO and O_3_. This part mainly discusses whether the annual average of the six criteria air pollutants has reached the Grade II standards, the annual variation of six of air quality levels, and the annual change of the non-attainment days (AQI > 100).

**Figure 1:**
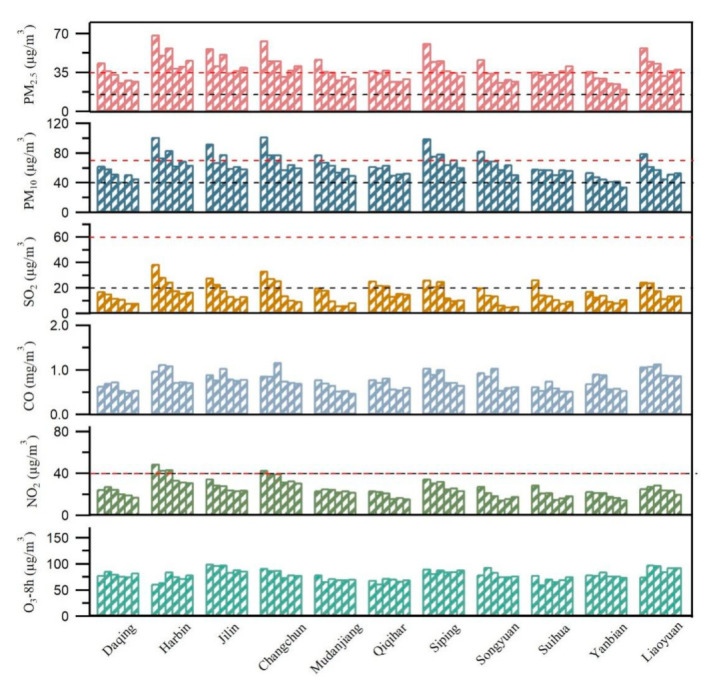
The annual average values of six pollutants from left to right in different cities represent the 2015–2020 period, respectively. The red dotted line represents Grade II standard and the black dotted line represents the Grade I standard according to the GB3059-2012.

The annual average of the six criteria air pollutants in HC was displayed in Figure 1. The red dotted line represents Grade II standards, and the black dotted line represents the Grade I standards. The inter-annual variation of PM_2.5_ showed a downward trend but the annual average in Harbin, Jilin, Changchun, and Liaoyuan still exceeded the Grade II standard (35 µg/m^3^). The annual average of PM_2.5_ in Daqing, Mudanjiang, Qiqihar, Siping, and Songyuan had years exceeding the Grade II standard before 2017 but reached the standard after 2018. It is worth mentioning that the annual average concentration of PM_2.5_ in Yanbian is lower than the standard in all years. However, the annual average concentration of PM_2.5_ in Suihua remained stable, but slightly exceeded the Grade II standard in 2019 (37.03 µg/m^3^) and 2020 (41.57 µg/m^3^). The inter-annual variation trend of PM_10_ is similar to PM_2.5_. The annual average concentration of PM_10_ in Daqing, Qiqihar, Suihua, and Yanbian was lower than the Grade II standard (70 µg/m^3^) in all years. Harbin, Changchun, Jilin, and Siping reached the Grade II standard after 2018. The annual average concentration of PM_10_ in Songyuan and Liaoyuan exceeded the standard only in 2015. The annual average of SO_2_ in all cities reached the Grade II standard (60 µg/m^3^) from 2015 to 2020. Only provincial capitals (Harbin and Changchun) failed to reach the Grade II standard (40 µg/m^3^) for NO_2_ before 2017. According to the comparison between the average annual concentration of pollutants and the Grade II standards, PM_2.5_ and PM_10_ are the main air pollutants in HC. It should be noted that SO_2_ and NO_2_ declined during the COVID-19 lockdown period compared to other years according to the Figures S1 and S2. These results are similar to the results of Kazuyuki. (2021) [33], which showed about 20% of NOx reduction was estimated in January and February 2020 for the whole of China using the global data assimilation.

**Figure 2:**
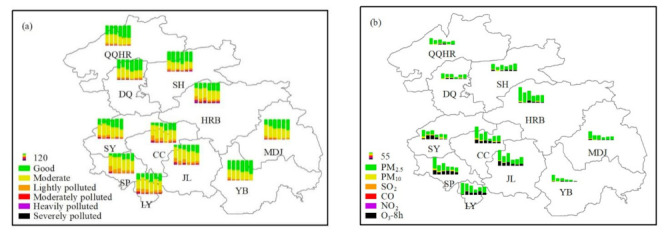
The six different polluted days and the major pollutants in different cities of HC. (**a**) The inter-annual change of six different polluted days in different cities of HC from 2015 to 2020. (**b**) The total non-attainment days (AQI > 100) and the major pollutants in the 11 cities of HC during 2015 to 2020.

Based on the AQI system, the air quality is divided into six levels. The inter-annual change of six different polluted days in different cities of HC from 2015 to 2020 is shown in Figure 2a. As shown in Figure 2a, it can be seen that the air quality in HC was gradually improving, mainly reflected in the obvious increase in the number of days with the level of “good”. The total non-attainment days (AQI > 100) and the major pollutants in the 11 cities of HC during 2015 to 2020 are shown in Figure 2b. The number of days with PM_2.5_ as the major pollutant was the largest in the whole year. In addition, the inter-annual variation of the total non-attainment days showed a trend of fluctuating decline.

### 3.2. The Major Pollutant

The proportion of the major pollutants during non-attainment periods in the 11 cities of HC is shown in Figure S3. The proportion of the days with PM_2.5_ as the major pollutant exceeded 50% in all cities. The proportion of PM_10_ as the major pollutant was higher in the western cities (Siping, Songyuan, and Daqing) than in other cities of HC. O_3_ was also one of the major pollutants but CO, NO_2_, and SO_2_ as the major pollutants rarely appeared in HC. In terms of the proportion of the major pollutants, the main pollutant in HC was PM_2.5_.

**Figure 3:**
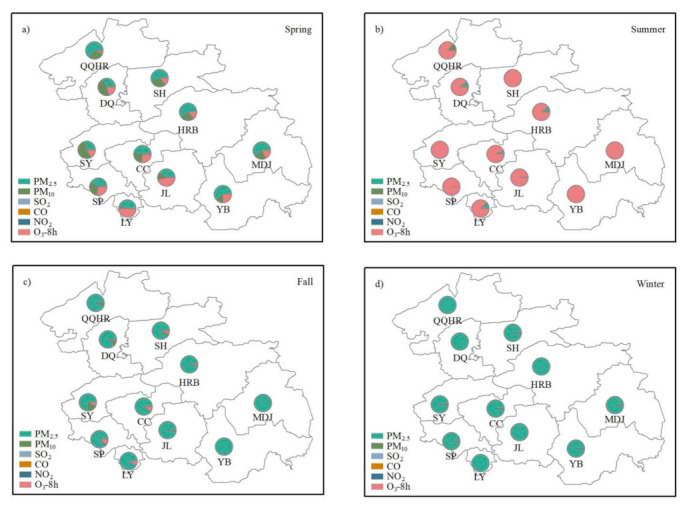
The proportion of the major pollutants in (**a**) Spring, (**b**) Summer, (**c**) Fall, and (**d**) Winter during non-attainment periods in the 11 cities of HC.

The proportion of the major pollutants in different seasons during non-attainment periods is shown in Figure 3a–d. In spring (From 1 March to 31 May), the proportion of PM_2.5_ as the major pollutant was usually the highest, which was displayed in Figure 3a. In Siping, Songyuan, and Daqing, the proportion of PM_10_ as the major pollutant was higher than in other cities. Based on our previous research [2], this may be influenced by the dust weather from Inner Mongolia, especially in spring. O_3_ was also one of the major pollutants in spring. The formation of O_3_ is influenced by precursor concentrations such as VOCs and photochemical reactions [5,34]. The photochemical reactions in spring and summer (From 1 June to 31 August) are usually higher than those in autumn and winter, which is the reason why the high proportion of O_3_ as the major pollutant often occurs in spring and summer. As shown in Figure 3b, the major pollutant in summer was O_3_. According to Figure 3c,d, the main pollutant in fall (From 1 September to 30 November) and winter (From 1 December to 28/29 February) was particulate matter, especially PM_2.5_.

### 3.3. The Pollution Characteristics of HC during Special Pollution Periods

#### 3.3.1. The Pollution Characteristics of Dust Weather

**Figure 4:**
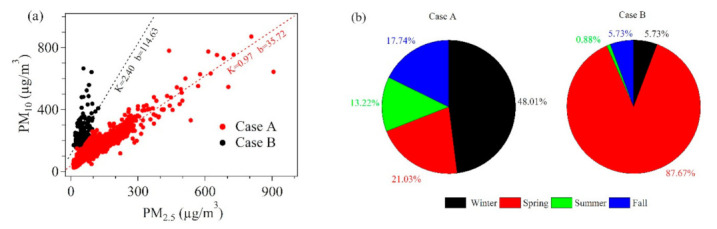
The scatter plots of the PM_10_ and PM_2.5_ was displayed in (**a**). Case B was divided under the condition that PM_10_ did not meet the daily Grade II standard (150 µg/m^3^) and the ratio of PM_10_/PM_2.5_ was greater than 2.7. The remaining part is Case A. (**b**) The pie charts show the percentages of the two Cases in different seasons.

Sandstorms are very common in northeast China, this has been reported by many studies [35,36]. The main pollutant brought by dust weather is particulate matter (especially PM_10_), and the ratio of PM_10_ to PM_2.5_ is a good indicator to distinguish dust weather. To discuss the influence of dust weather, scatter plots of PM_10_ and PM_2.5_ were made in the left part of Figure 4. It is clearly seen that the red dots have a significantly higher ratio of PM_10_/PM_2.5_. When discussing the impact of dust weather, the data we select is only non-attainment data (Total 3570 points). Case B was divided under the condition that PM_10_ did not meet the daily Grade II standard (150 µg/m^3^) and the ratio of PM_10_/PM_2.5_ was greater than 2.7. The rest of the polluted weather was Case A. In this way, Case B which was greatly affected by dust can be segmented to discuss the pollution characteristics of the two Cases. The pie charts on the right of Figure 4 showed the percentages of the two Cases in different seasons. According to the pie charts, the data of Case B mainly comes from the spring period, which also proves that our division is relatively accurate.

**Figure 5:**
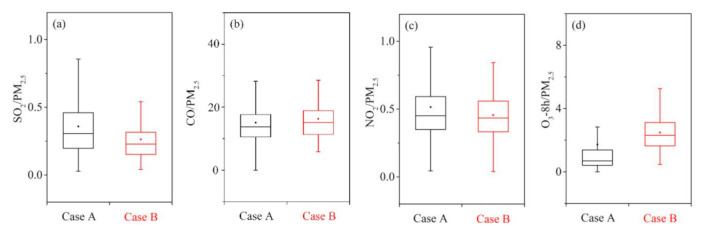
The ratio characteristics of air pollutants between Case A and B. (**a**) SO_2_/PM_2.5_, (**b**) CO/PM_2.5_, (**c**) NO_2_/PM_2.5_, and (**d**) O_3_/PM_2.5_.

According to the classification of Case A and B, the ratio of SO_2_/PM_2.5_, CO/PM_2.5_, NO_2_/PM_2.5_, and O_3_/PM_2.5_ is discussed in Figure 5a–d. The SO_2_/PM_2.5_ ratio of Case B is slightly lower than that of Case A according to Figure 5a. As shown in Figure 5b,c, there are no significant differences in CO/PM_2.5_ and NO_2_/PM_2.5_ ratios between Case A and B. As shown in Figure 5d, the ratio of O_3_/PM_2.5_ in Case B is significantly higher than that of Case A, which seems to be an uncommon phenomenon because the contribution of dust to the O_3_ generated by the secondary reaction is limited. The formation of O_3_ is influenced by precursor concentrations such as VOCs and photochemical reactions [5,34]. According to the characteristics of Case B, almost all data are taken from spring. Therefore, the reason for the high O_3_/PM_2.5_ in Case B should be the strong photochemical reactions in spring that lead to the production of O_3_.

#### 3.3.2. The Pollution Characteristics of Open Biomass Burning (OBB)

**Figure 6:**
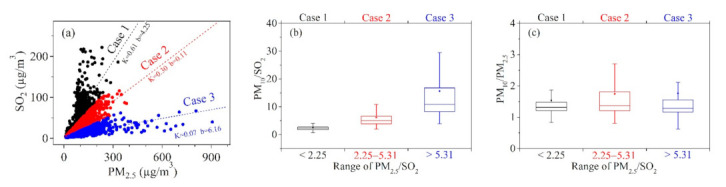
(**a**) The scatter plots of the PM_2.5_ and SO_2_. Case1, Case2, and Case3 represent PM_2.5_/SO_2_ ratios ranging from <2.25, 2.25–5.31, and >5.31, respectively. (**b**) The PM_10_/SO_2_ ratio of Case 1, Case 2, and Case 3. (**c**) The PM_10_/PM_2__.5_ ratio of Case 1, Case 2, and Case 3.

The grain yield in HC is very competitive in China [37]. After harvest, the crop residue was usually burned directly by farmers to ensure the next spring tillage, which leads to a lot of OBB phenomena. In Beijing, heavy pollution can be caused by adverse diffusion meteorological conditions [12] or the growth of secondary aerosols [38] rather than OBB. Regional transport (the western part of Jiangsu province) is one of the main reasons of the haze period in the YRD region [39,40]. During the haze period in the PRD region, the descending motion is prevailing in the planetary boundary layer (PBL) [41]. However, heavy haze events caused by large-scale OBB are common in HC, but rarely occur in BTH, YRD, and PRD regions which have unique episodes of HC. According to published reports, the emission factors of PM_2.5_ from biomass combustion are much higher than SO_2_ [42,43,44]. According to the results of Cao et al. (2008) [42], the ratio of PM_2.5_/SO_2_ produced by stove combustion is 132.75 for corn straw and 34.89 for rice straw, respectively. The PM_2.5_/SO_2_ ratio of agricultural residues was 10.5 which was measured by Andreae (2019) [43]. Xu et al. (2019) also summarized the emission factors of various crops (corn, rice, and wheat), and the PM_2.5_/SO_2_ ratio in these emission factors was all greater than 10 [44]. According to previous stove combustion experiments, the PM_2.5_/SO_2_ ratio produced by biomass combustion is relatively high (higher than 10). In the observation experiment, the PM_2.5_/SO_2_ ratio also increased when there were more fire points. In addition, in previous studies, PM_2.5_/SO_2_ frequency distribution presents a bimodal distribution when the concentration of PM_2.5_ is higher than 115 µg/m^3^ [2]. Therefore, PM_2.5_/SO_2_ is effective in distinguishing OBB, especially on heavy pollution days. Based on previous research results, this paper divided three Cases (Case 1, Case 2, and Case 3) by the PM_2.5_/SO_2_ ratio, which was displayed in Figure 6a. The contribution of OBB to Case 1, Case 2, and Case 3 is gradually increasing. It is worth noting that the heavy pollution with PM_2.5_ greater than 500 µg/m^3^ all occurred in Case 1, which had a relatively large contribution from OBB. This phenomenon is a unique characteristic of HC.

The ratio of PM_10_/SO_2_ and PM_10_/PM_2.5_ for Case 1, Case 2, and Case 3 was displayed in Figure 6b,c, respectively. In Case 3, the contribution of biomass combustion is relatively large, the PM_10_/SO_2_ ratio is the highest, but the PM_10_/PM_2.5_ ratio is not. This indicates that the concentration of particulate matter (PM_2.5_ and PM_10_) increases during OBB periods, but the increase of the fine particulate (PM_2.5_) is more evident than the coarse particulate (PM_10_).

**Figure 7:**
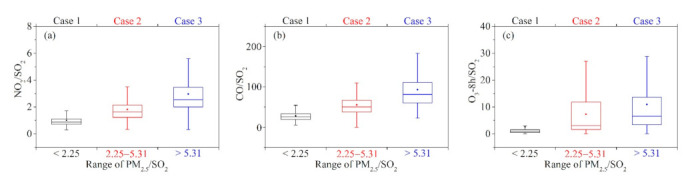
The ratio characteristics of air pollutants between Case 1, Case 2, and Case 3. (**a**) NO_2_/SO_2_, (**b**) CO/SO_2_, (**c**) O_3_-8 h/SO_2_.

In Figure 7a–c, the ratio of NO_2_/SO_2_, CO/SO_2_, and O_3_-8 h/SO_2_ in three Cases was discussed. The ratios of NO_2_/SO_2_ and CO/SO_2_ showed an increasing trend from Case 1 to Case 3. Nitrogen fertilizer is one of the main fertilizers of crops. It is normal for a relatively high NO_2_/SO_2_ ratio to appear in Case 3. Biomass combustion, especially incomplete combustion, produces a large amount of CO [22,27]. According to the results of Cheng et al. (2020), the ratio of EC/CO (the tracer to identify flaming and smoldering) is small in the Case that contributes more to OBB which indicates the OBB in HC tends to incomplete combustion [8]. Therefore, the increasing trend of CO/SO_2_ ratio from Case 1 to Case 3 is credible. Compared with Case 1, the ratio of O_3_-8 h/SO_2_ in Case 2 and Case 3 are also higher. This may be due to the large amount of VOCs emitted by OBB, and the increase of ozone precursors which leads to the increase in the concentration of O_3_.

### 3.4. The Correlation between 11 Cities and the Coordinated Control of PM_2.5_ and O_3_

#### 3.4.1. The Pearson Correlation Coefficients between 11 Cities

**Figure 8:**
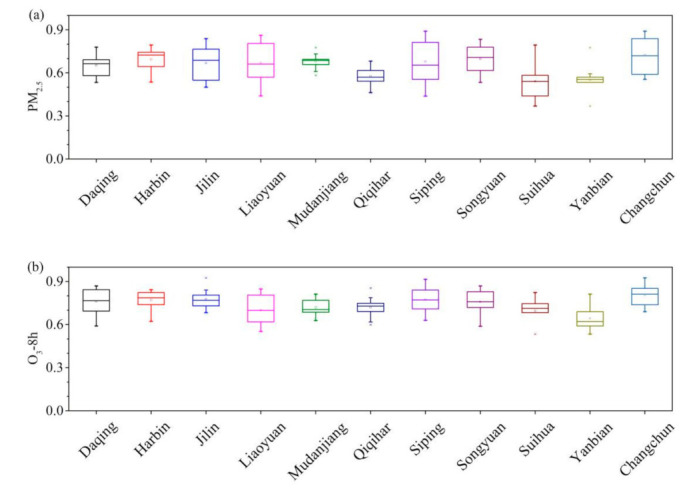
The box plot of Pearson correlation coefficient of (**a**) PM_2.5_ and (**b**) O_3_-8 h. for each city with other cities in HC.

This part mainly discusses the Pearson correlation coefficients of various pollutants in 11 cities of HC. Tables S1–S7 represents the Pearson correlation coefficients of AQI, PM_2.5_, PM_10_, SO_2_, CO, NO_2_, and O_3_-8 h, respectively. In order to clearly understand the Pearson correlation coefficients in HC, the different pollutants’ correlation coefficients of each city and the other 10 cities are made into a box plot. The Pearson correlation coefficients of PM_2.5_, O_3_-8 h, AQI, PM_10_, SO_2_, CO, and NO_2_ in each city were shown in Figure 8a,b and Figure S4a–e, respectively. The Pearson correlation coefficients of all pollutants in HC are high. The regional Pearson correlation coefficients of secondary pollutant (O_3_-8 h) are higher than that of primary pollutant (PM_2.5_).

#### 3.4.2. The Coordinated Control of PM_2.5_ and O_3_

**Figure 9:**
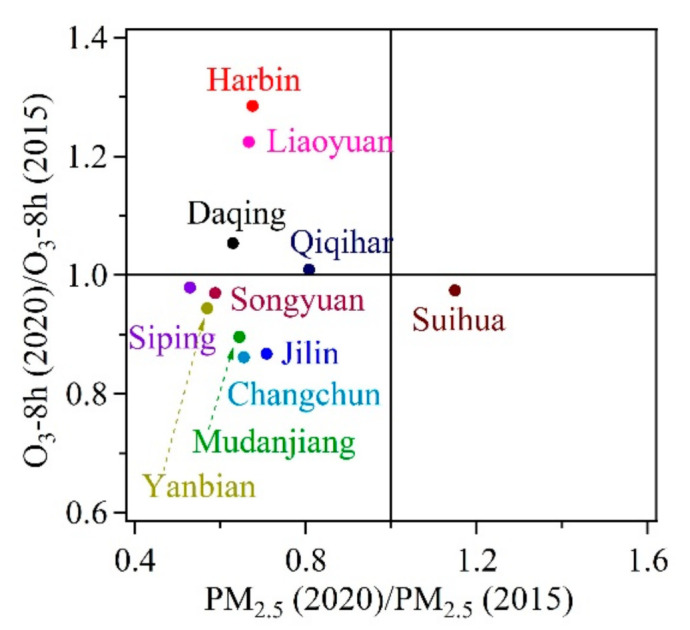
The proportion of the major pollutants during non-attainment periods in the 11 cities of HC.

A quadrant graph is made by taking the ratio of PM_2.5_ in 2020 to PM_2.5_ in 2015 as the X-axis and the ratio of O_3_ in 2020 to O_3_ in 2015 as the Y-axis, which is displayed in Figure 9. Yanbian, Changchun, Jilin, Mudanjiang, Songyuan, and Siping showed a double downward trend in PM_2.5_ and O_3_. PM_2.5_ in Harbin, Liaoyuan, Daqing, and Qiqihar shows a downward trend while O_3_ shows an upward trend. Therefore, these cities should be paid more attention to the coordinated control of PM_2.5_ and O_3_. In Suihua, O_3_ shows a downward trend, but PM_2.5_ shows an upward trend. The city still needs to give priority to controlling the growth of PM_2.5_.

## 4. Conclusions

The air quality in HC was gradually improving, mainly reflected in the obvious increase in the number of days with the level of “good”. The inter-annual variation of the total non-attainment days (AQI > 100) showed a trend of fluctuating decline. In spring, the major pollutants in HC were PM_2.5_, PM_10_, and O_3_. In summer, autumn, and winter, the major pollutant was O_3_, PM_2.5_, and PM_2.5_, respectively. The dust weather mainly contributes to particulate pollutants but has limited contribution to gaseous pollutants. OBB has a certain contribution to all kinds of pollutants, among which the contribution to PM_2.5_ is higher than PM_10_. The correlation between pollutants in HC is high. The regional Pearson correlation coefficients of secondary pollutant (O_3_-8 h) are higher than that of primary pollutant (PM_2.5_). The cities of Harbin, Liaoyuan, Daqing, and Qiqihar should be paid more attention regarding the coordinated control of PM_2.5_ and O_3_.

## Figures and Tables

**Table 1:** The individual air quality index (AQI) and corresponding pollutant concentration limits.

IAQI	The Concentration Limits of Different Pollutants					
	The 24-h Average Concentration of PM_2.5_ (μg/m^3^)	The 24-h Average Concentration of PM_10_ (μg/m^3^)	The 24-h Average Concentration of SO_2_ (μg/m^3^)	The 24-h Average Concentration of CO (mg/m^3^)	The 24-h Average Concentration of NO_2_ (μg/m^3^)	The 8-h Moving Average Concentration of O_3_ (μg/m^3^)
0	0	0	0	0	0	0
50	35	50	50	2	40	100
100	75	150	150	4	80	160
150	115	250	475	14	180	215
200	150	350	800	24	280	265
300	250	420	1600	36	565	800
400	350	500	2100	48	750	*
500	500	600	2620	60	940	*

* The IAQI of O_3_ is not calculated when the 8-h moving average concentration of O_3_ is higher than 800 μg/m^3^.

## Data Availability

The mass concentration data of six air pollutants was downloaded from the China Air Quality Online Monitoring and Analysis Platform (https://www.aqistudy.cn/, accessed on 16 December 2021).

## Supplementary Materials

The following are available online at https://www.mdpi.com/article/10.3390/toxics9120357/s1, S1: The detection methods of the six criteria air pollutants. Figure S1: The concentration of six pollutants in HC from 2015 to 2020 in January. From left to right represent the years from 2015 to 2020, respectively, Figure S2: The concentration of six pollutants in HC from 2015 to 2020 in February. From left to right represent the years from 2015 to 2020, respectively, Figure S3: The proportion of the major pollutants during non-attainment periods in the 11 cities of HC, Figure S4: The box plot of Pearson correlation coefficient of different pollutants for each city with other cities in HC. (**a**) AQI, (**b**) PM_10_, (**c**) SO_2_, (**d**) CO, and (**e**) NO_2_, Table S1: The Pearson Correlation of AQI in HC, Table S2: The Pearson Correlation of PM_2.5_ in HC, Table S3: The Pearson Correlation of PM_10_ in HC, Table S4: The Pearson Correlation of SO_2_ in HC, Table S5: The Pearson Correlation of CO in HC, Table S6: The Pearson Correlation of NO_2_ in HC, Table S7: The Pearson Correlation of O_3_-8 h in HC.
