# Supplementary material for: Air Quality in the Harbin-Changchun Metropolitan Area in Northeast China: Unique Episodes and New Trends
Source: Toxics. 2021 Dec 17;9(12):357. doi: 10.3390/toxics9120357 (PMC8707320; doi:10.3390/toxics9120357)
Supplement: Supplementary file 1 [file toxics-09-00357-s001.zip › toxics-1482443-supplementary.pdf]

toxics MDPI

Air quality in the Harbin-Changchun metropolitan area in Northeast China: unique episodes and new trends

Yulong Wang, Youwen Sun, Gerong Zhao and Yuan Cheng

S1. The detection methods of the six criteria air pollutants

According to the HJ655-2013, the concentrations of $PM_{2.5}$ and PM_{10} are measured by the micro oscillating balance method and the β absorption method, respectively. According to the HJ193-2013, the ultraviolet fluorescence method, chemiluminescence method, and UV-spectrophotometry method are used to measure the mass concentration of SO_2, NO_2, and O_3, respectively. Besides, the gas filter correlation infrared absorption method and the non-dispersive infrared absorption method are used to measure the CO concentration.

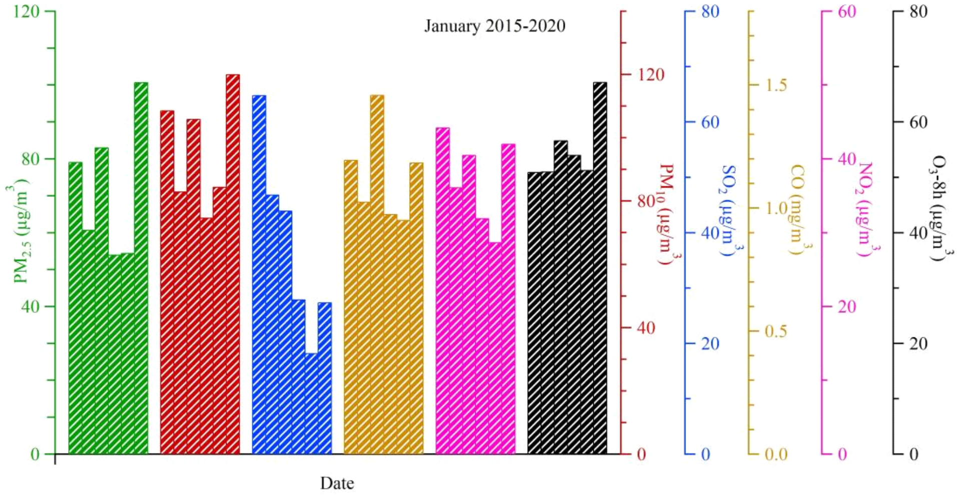

Figure S1. The concentration of six pollutants in HC from 2015 to 2020 in January. From left to right represent the years from 2015 to 2020, respectively.

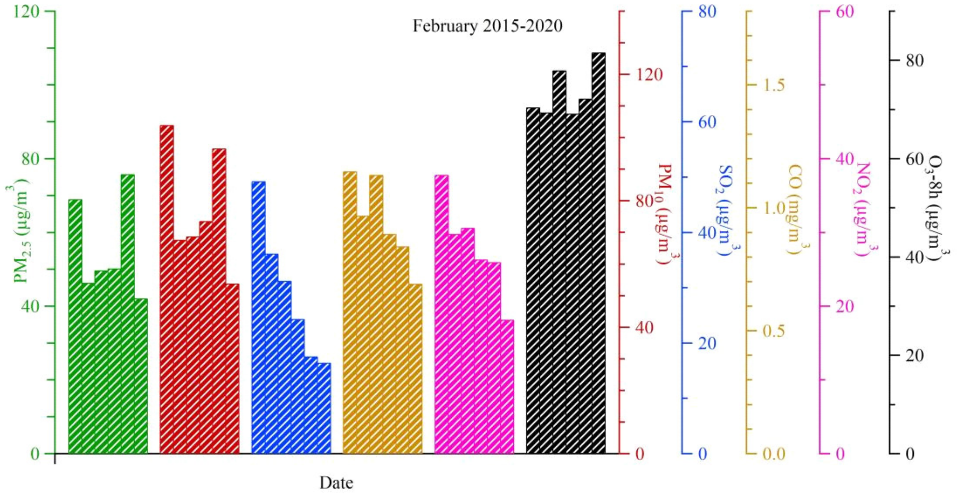

Figure S2. The concentration of six pollutants in HC from 2015 to 2020 in February. From left to right represent the years from 2015 to 2020, respectively.

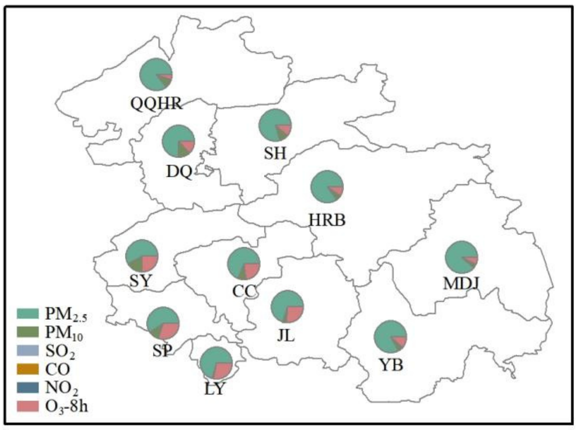

Figure S3. The proportion of the major pollutants during non-attainment periods in the 11 cities of HC.

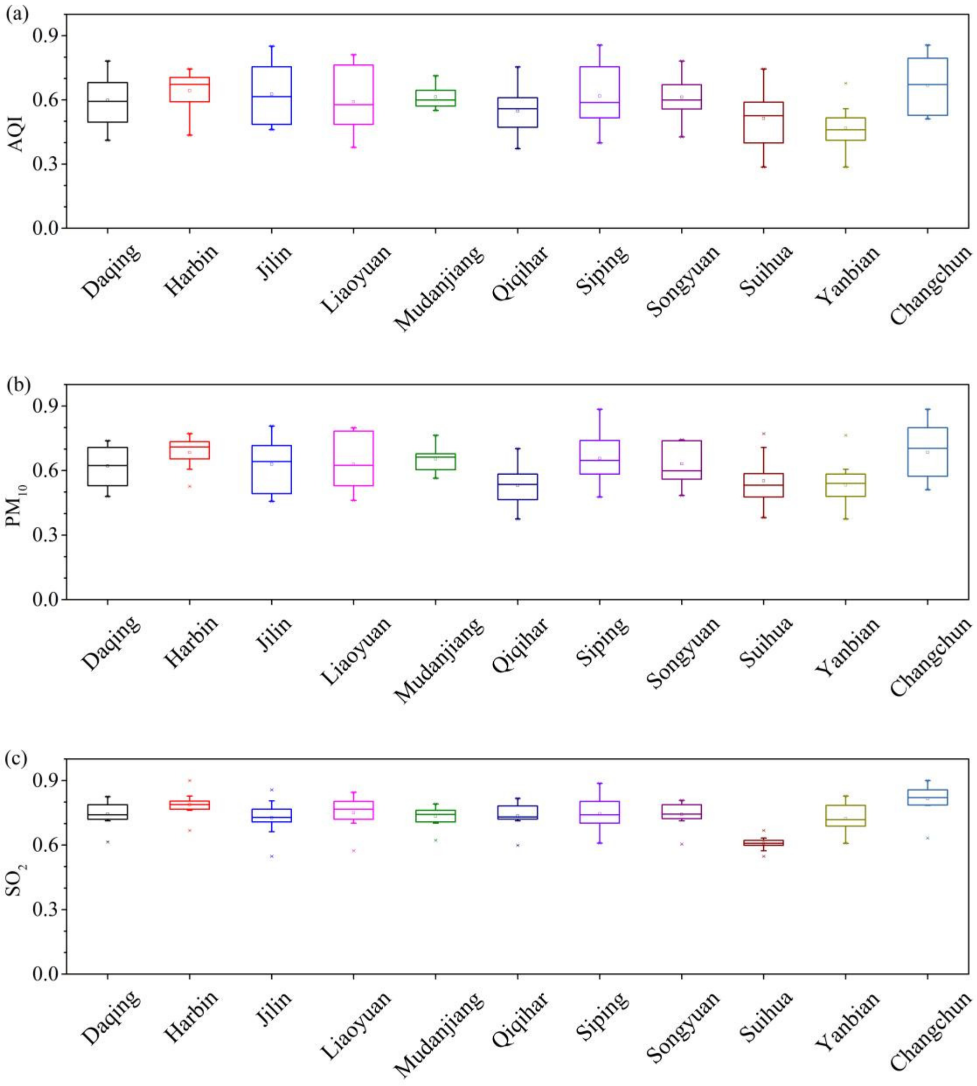

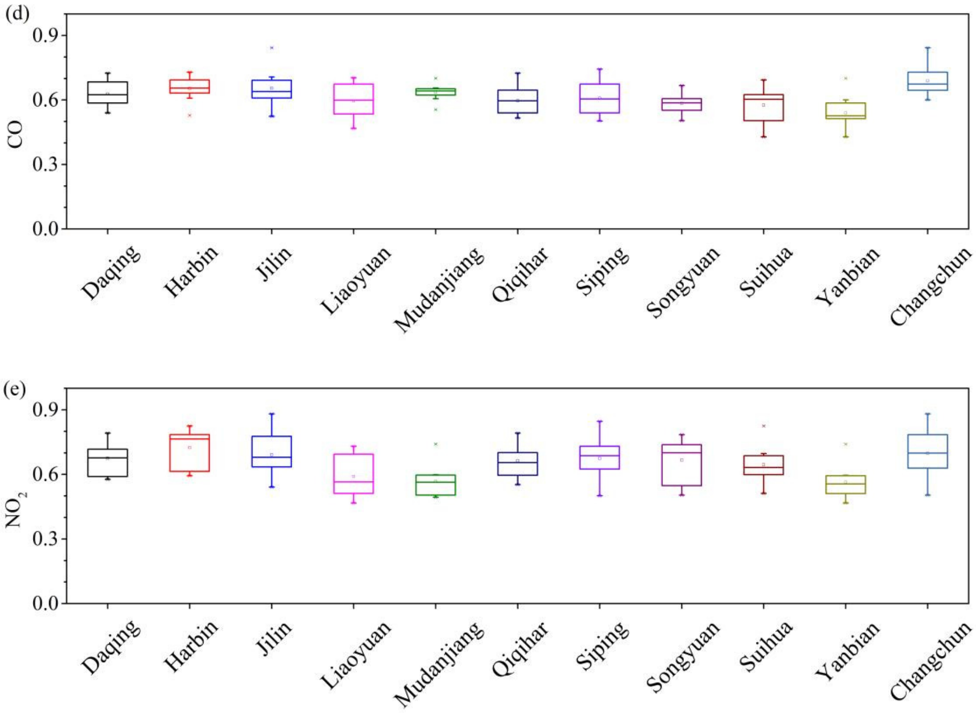

Figure S4. The box plot of Pearson correlation coefficient of different pollutants for each city with other cities in HC. (**a**) AQI, (**b**) PM_{10}, (**c**) SO_2, (**d**) CO, and (**e**) NO_2.

Table S1. The Pearson Correlation of AQI in HC.

Cities	Daqing	Har-bin	Jilin	Liaoyuan	Mu-dan-jiang	Qiqi-har	Siping	Song-yuan	Sui-hua	Yanbian	Changchun
**Daqing**	1										
**Harbin**	0.681**	1									
**Jilin**	0.486**	0.675**	1								
**Liaoyuan**	0.496**	0.584**	0.763**	1							
**Mudanjiang**	0.607**	0.713**	0.645**	0.571**	1						
**Qiqihar**	0.754**	0.634**	0.477**	0.441**	0.592**	1					
**Siping**	0.524**	0.591**	0.755**	0.811**	0.584**	0.472**	1				
**Songyuan**	0.782**	0.670**	0.586**	0.589**	0.557**	0.610**	0.671**	1			
**Suihua**	0.668**	0.745**	0.461**	0.378**	0.551**	0.589**	0.399**	0.540**	1		
**Yanbian**	0.411**	0.435**	0.559**	0.486**	0.678**	0.372**	0.516**	0.427**	0.286**	1	
**Changchun**	0.578**	0.705**	0.851**	0.795**	0.641**	0.528**	0.856**	0.702**	0.512**	0.511**	1

** Correlation is significant at the 0.01 level.

Table S2. The Pearson Correlation of $PM_{2.5}$ in HC.

Cities	Daqing	Har-bin	Jilin	Liaoyuan	Mu-dan-jiang	Qiqi-har	Siping	Song-yuan	Sui-hua	Yanbian	Changchun
**Daqing**	1										
**Harbin**	0.762**	1									
**Jilin**	0.549**	0.716**	1								
**Liaoyuan**	0.581**	0.644**	0.805**	1							
**Mudanjiang**	0.692**	0.732**	0.691**	0.679**	1						

**Qiqihar**	0.682**	0.622**	0.542**	0.531**	0.610**	1					
**Siping**	0.620**	0.649**	0.765**	0.854**	0.658**	0.550**	1				
**Songyuan**	0.779**	0.744**	0.686**	0.729**	0.683**	0.617**	0.813**	1			
**Suihua**	0.657**	0.794**	0.500**	0.440**	0.583**	0.547**	0.438**	0.534**	1		
**Yanbian**	0.534**	0.536**	0.594**	0.570**	0.776**	0.463**	0.555**	0.553**	0.369**	1	
**Changchun**	0.671**	0.744**	0.839**	0.862**	0.694**	0.590**	0.891**	0.834**	0.555**	0.563**	1

** Correlation is significant at the 0.01 level.

Table S3. The Pearson Correlation of PM_{10} in HC.

Cities	Daqing	Harbin	Jilin	Liaoyuan	Mudanjiang	Qiqihar	Siping	Songyuan	Suihua	Yanbian	Changchun
**Daqing**	1										
**Harbin**	0.734**	1									
**Jilin**	0.484**	0.697**	1								
**Liaoyuan**	0.530**	0.654**	0.783**	1							
**Mudanjiang**	0.654**	0.723**	0.678**	0.670**	1						
**Qiqihar**	0.702**	0.606**	0.457**	0.465**	0.564**	1					
**Siping**	0.593**	0.662**	0.716**	0.783**	0.633**	0.490**	1				
**Songyuan**	0.739**	0.731**	0.580**	0.594**	0.604**	0.560**	0.740**	1			
**Suihua**	0.708**	0.772**	0.493**	0.461**	0.586**	0.584**	0.477**	0.536**	1		
**Yanbian**	0.480**	0.527**	0.606**	0.554**	0.764**	0.375**	0.584**	0.484**	0.381**	1	
**Changchun**	0.593**	0.736**	0.807**	0.799**	0.671**	0.511**	0.885**	0.743**	0.528**	0.574**	1

** Correlation is significant at the 0.01 level.

Table S4. The Pearson Correlation of SO_2 in HC.

Cities	Daqing	Harbin	Jilin	Liaoyuan	Mudanjiang	Qiqihar	Siping	Songyuan	Suihua	Yanbian	Changchun
**Daqing**	1										
**Harbin**	0.681**	1									
**Jilin**	0.486**	0.675**	1								
**Liaoyuan**	0.496**	0.584**	0.763**	1							
**Mudanjiang**	0.607**	0.713**	0.645**	0.571**	1						
**Qiqihar**	0.754**	0.634**	0.477**	0.441**	0.592**	1					
**Siping**	0.524**	0.591**	0.755**	0.811**	0.584**	0.472**	1				
**Songyuan**	0.782**	0.670**	0.586**	0.589**	0.557**	0.610**	0.671**	1			
**Suihua**	0.668**	0.745**	0.461**	0.378**	0.551**	0.589**	0.399**	0.540**	1		
**Yanbian**	0.411**	0.435**	0.559**	0.486**	0.678**	0.372**	0.516**	0.427**	0.286**	1	
**Changchun**	0.578**	0.705**	0.851**	0.795**	0.641**	0.528**	0.856**	0.702**	0.512**	0.511**	1

** Correlation is significant at the 0.01 level.

Table S5. The Pearson Correlation of CO in HC.

Cities	Daqing	Harbin	Jilin	Liaoyuan	Mudanjiang	Qiqihar	Siping	Songyuan	Suihua	Yanbian	Changchun
**Daqing**	1										
**Harbin**	0.689**	1									
**Jilin**	0.609**	0.707**	1								
**Liaoyuan**	0.540**	0.632**	0.685**	1							
**Mudanjiang**	0.652**	0.639**	0.656**	0.623**	1						

**Qiqihar**	0.725**	0.608**	0.616**	0.518**	0.646**	1					
**Siping**	0.546**	0.643**	0.692**	0.674**	0.606**	0.540**	1				
**Songyuan**	0.606**	0.667**	0.598**	0.575**	0.556**	0.552**	0.604**	1			
**Suihua**	0.639**	0.693**	0.623**	0.535**	0.625**	0.584**	0.502**	0.504**	1		
**Yanbian**	0.586**	0.528**	0.524**	0.468**	0.701**	0.516**	0.528**	0.513**	0.428**	1	
**Changchun**	0.684**	0.729**	0.843**	0.703**	0.645**	0.653**	0.744**	0.664**	0.622**	0.600**	1

** Correlation is significant at the 0.01 level.

Table S6. The Pearson Correlation of NO_2 in HC.

**Cities**	**Daqing**	**Harbin**	**Jilin**	**Liaoyuan**	**Mudanjiang**	**Qiqihar**	**Siping**	**Songyuan**	**Suihua**	**Yanbian**	**Changchun**
**Daqing**	1										
**Harbin**	0.789**	1									
**Jilin**	0.665**	0.777**	1								
**Liaoyuan**	0.577**	0.614**	0.694**	1							
**Mudanjiang**	0.586**	0.597**	0.541**	0.494**	1						
**Qiqihar**	0.792**	0.752**	0.647**	0.553**	0.595**	1					
**Siping**	0.673**	0.722**	0.784**	0.731**	0.501**	0.650**	1				
**Songyuan**	0.717**	0.785**	0.738**	0.548**	0.504**	0.701**	0.700**	1			
**Suihua**	0.681**	0.825**	0.635**	0.512**	0.599**	0.687**	0.625**	0.697**	1		
**Yanbian**	0.590**	0.594**	0.552**	0.467**	0.741**	0.596**	0.511**	0.520**	0.560**	1	
**Changchun**	0.685**	0.785**	0.881**	0.713**	0.517**	0.660**	0.847**	0.753**	0.629**	0.504**	1

** Correlation is significant at the 0.01 level.

Table S7. The Pearson Correlation of O_3-8h in HC.

**Cities**	**Daqing**	**Harbin**	**Jilin**	**Liaoyuan**	**Mudanjiang**	**Qiqihar**	**Siping**	**Songyuan**	**Suihua**	**Yanbian**	**Changchun**
**Daqing**	1										
**Harbin**	0.843**	1									
**Jilin**	0.746**	0.770**	1								
**Liaoyuan**	0.694**	0.702**	0.806**	1							
**Mudanjiang**	0.688**	0.739**	0.806**	0.628**	1						
**Qiqihar**	0.854**	0.787**	0.691**	0.599**	0.700**	1					
**Siping**	0.756**	0.786**	0.840**	0.848**	0.709**	0.719**	1				
**Songyuan**	0.869**	0.829**	0.768**	0.729**	0.654**	0.749**	0.821**	1			
**Suihua**	0.776**	0.823**	0.683**	0.553**	0.686**	0.746**	0.706**	0.719**	1		
**Yanbian**	0.590**	0.623**	0.730**	0.619**	0.812**	0.618**	0.629**	0.588**	0.533**	1	
**Changchun**	0.811**	0.812**	0.925**	0.830**	0.769**	0.738**	0.916**	0.852**	0.726**	0.690**	1

** Correlation is significant at the 0.01 level.
